# Investigating factors underlying why householders remain in at-risk areas during bushfire disaster in Australia

**DOI:** 10.1016/j.heliyon.2024.e29727

**Published:** 2024-04-16

**Authors:** Olufisayo Adedokun, Temitope Egbelakin, Willy Sher, Thayaparan Gajendran

**Affiliations:** School of Architecture and Built Environment, University of Newcastle, University Drive, Callaghan, 2308, NSW, Australia

**Keywords:** Australia, Black summer, Bushfires, Disasters, Fatality, Householders

## Abstract

Although most homes lack design and construction features to withstand bushfires, there is a growing trend of householders relocating to bushfire-prone areas. Notably, many bushfire-related fatalities have occurred within proximity, specifically within 100 m of bushland. Therefore, this paper explores the factors that drive householders to remain in at-risk areas, despite the imminent threat of bushfires. Thirty semi-structured interviews were conducted with participants residing in the southeastern region of New South Wales (NSW). The interviews were recorded, transcribed using Otter.AI, and subjected to thematic analysis using NVivo 12 Pro. The findings shed light on why certain householders decided to stay on their properties during the catastrophic 2019/2020 bushfires. Upon analysis, we uncovered thirty-six distinct factors that underlie householders’ choices to remain during bushfires. These factors were categorized into nine groups: protection-related, attitude-related, information-related, operation-related, road-related, shelter-related, finance-related, health-related, and rebuilding-related factors. The study underscores the importance of understanding gender-based differences and pet ownership in bushfire evacuation decisions, emphasizing the need for tailored communication strategies. These findings hold several important implications for research and practice regarding early self-evacuation from bushfires.

## 1 Introduction

Bushfires are annual reoccurring events which can have unimaginable consequences, including loss of lives, well-being, and economic costs [1]. Despite the risks associated with bushfires, people tend to live in urban-bush interfaces because of the attractiveness of living in a natural environment. In addition, some people migrate to urban bushland regions because it offers more affordable housing and less congestion [2]. However, this is usually done without taking adequate cognizance of the risks associated with exposure to bushfires. For instance, 35 fatalities occurred during the 2019/2020 Australia bushfires, aside from 429 smoke-related deaths [3,4]. With the predictions of more frequent destructive events like the 2019/2020 bushfires, more fatalities and increasing survivors' vulnerability to several health-related problems are imminent because of climate change [5]. The risk is exacerbated because 90 % of homes in the wildland-urban interface (WUI) regions are not bushfire resilient [6]. Despite the emergency agencies' advocacy for early self-evacuation from at-risk communities, not leaving, and late evacuation from locations within 100 m of bushfire-prone areas led to fatalities in the past [7]. Further, people in these communities underestimate the risks associated with bushfires and are ill-prepared [8]. Therefore, timely decision-making is essential for householders’ survival in communities where evacuation is either mandatory or voluntary.

Emergency agencies advocate for early evacuation as the most adaptive and ideal protection action because it ensures the safety of lives if undertaken well before the threat becomes imminent, especially in severe bushfires [9]. However, early evacuation often depends on the bushfires, individual characteristics, preparedness levels and community perceptions of risk and responsibility. While planning and preparedness levels can make or mar decision-making and actions during bushfire hazards, varying protective action alternatives could complicate the pre-decision and decision-making processes [10]. For instance, various bushfire response actions include evacuation, staying and defending property and sheltering in place [11] or a combination of the preceding [10]. Despite the levels of information about bushfire response actions, Australia is one of the most bushfire-prone places in the world, where high death rates are recorded [12]. Moreover, millions of hectares have been burnt and many homes destroyed during fire seasons [13]. Furthermore, 40 % of natural hazard deaths in Australia are associated with bushfires [7], because homeowners are increasingly relocating to high-risk areas. In response to these deaths, the Australian bushfire policy (prepare, stay, and defend or leave early) was revised to recommend leaving early as the safest option [14]. Despite the revision and emphasis on early evacuation, some wildland-urban interface (WUI) residents are still not yielding to the early evacuation advice.

In these perilous zones, where the threat of bushfires looms large, a critical issue surfaces: numerous fatalities have tragically occurred within a mere 100 m of bushland. This troubling scenario prompts a pivotal research question that guides our inquiry. It stems from a profound observation, grounded in previous studies, that many residents in these bushland areas consistently opt to stay on their properties when bushfires strike. It is a curious choice, considering the potential for catastrophic outcomes. So, what drives these individuals to make such decisions in the face of looming danger? To unravel this enigma, this paper aims to investigate factors underlying why householders remain in at-risk areas during bushfires. Only through this comprehensive understanding can we develop effective strategies that encourage timely evacuation when confronted with the ominous specter of bushfire threats.

## 2 Literature review

### 2.1 Bushfires in Australia

Bushfires bring damage and disruption to infrastructure and activities, humans, and livestock. They are driven by the weather conditions, availability of fuel, and the presence of ignition. While the ignition source could be natural, human-made ignition is also possible. One of the significant causes of bushfire ignition is natural lightning [15]. For example, the state of Victoria experienced enormous bushfire damage due to natural lightning [16]. Human activities, such as burning of agricultural waste, incorrect equipment handling (negligent or accidental) and faulty overhead powerlines have been linked to several bushfires. Therefore, human activities in forested landscapes increase the susceptibility of humans and their homes to bushfire attacks [17,18].

The sparks from faulty overhead powerlines have also caused vegetation to ignite on many occasions leading to bushfires [19]. For example, the Waroona Perth 2016 fire in Western Australia and the 2019/2020 bushfires in South-Eastern Australia were triggered by ignitions from faulty powerlines [[20], [21], [22]]. The inquiry into the NSW 2019/2020 bushfire also attributed its cause to climate change [20]. Besides, the 2019/2020 bushfire season was characterized by several fire-generated thunderstorms. For example, this season experienced 30 fire-generated thunderstorms compared to 60 in almost 19 years between the 1980s till late 2018/2019 [20]. The damaged power lines gave rise to power outages, communication breakdowns in some locations and prevented residents from contacting family, friends and receiving emergency alerts [23]. The implication is that bushfires have corresponding impacts on lives and livelihoods in Australia.

### 2.2 Bushfire impacts in Australia

Bushfires can inflict a multitude of adverse effects on human lives and the things people value. Loss of life is one impact of bushfire, and it has accounted for more than 825 deaths in Australia over 110 years of bushfire seasons [24,25]. In addition to the loss of life, many homes were also destroyed and damaged by fires. The significant home losses are consequent upon embers that landed before or after the actual fire front passed through [26]. Some of the short-term impacts of bushfire include extraordinary sound, smell, unpredictability, and inability to breathe. During this period, air quality is affected, leading to smoke inhalation [27]. Wildfire smoke and ash can affect human health, transport, agriculture, and biodiversity far from the fire itself [28]. The long-term impacts of fires on children, firefighters and the community include weather phobias, especially intense anxiety when there are strong, hot winds [29].

The survivors of bushfires frequently present with mental health symptoms. For example, following the 2009 Victorian bushfire in Australia, a significant number of the survivors reported post-traumatic stress disorder, severe psychological distress and depression [30]. The survivors of this bushfire event are still experiencing severe mental health-related problems like depression, anxiety, nightmares, and somatic complaints [29,31]. Furthermore, health costs, opportunity costs for volunteer firefighters, fixed costs for firefighting services and impacts on ecosystem services all result from bushfires [32]. In addition, bushfire disasters are associated with an increased prevalence of financial impacts on individuals and governments as homes and communities need to be rebuilt asides from the post-disaster clean-up [31]. As bushfire disasters become more prevalent in Australia, these impacts look set to worsen as more people are affected.

According to de Vet and Eriksen [33], although many households are underinsured, home and contents insurance is fundamental to household and community resilience against bushfire disasters. Approximately $337 million per year was estimated as the total economic cost of bushfires in Australia. With an annual forecast growth rate of 2.2 %, the annual total economic cost of bushfires in Australia is projected to reach $800 million by 2050 [32,34]. Arising from the 2019/2020 bushfire seasons, more than 23,362 claims for fires across New South Wales, Queensland, South Australia, and Victoria filed between November 8 and February 14, 2020 [35]. In addition, the 2019/2020 bushfire season cost more than $1.7 million in insurance losses [36]. According to the Centre for Disaster Philanthropy [35], with a 1 % drop in Australia's gross domestic product, a corresponding estimated figure of $20 billion could be wiped from the economy. This is consequent upon damages to tourism, agriculture, property, livestock, factories, and other sectors of the economy [35].

### 2.3 Bushfire fatalities in Australia

**Fig. 1:**
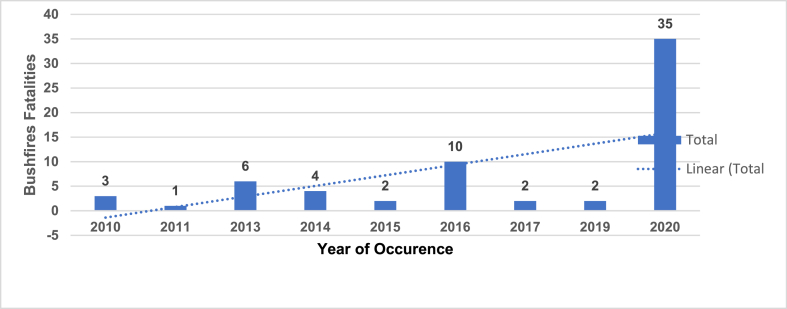
Fatality rate in Australia.

Source: Risk Frontiers [23]. * from 2010 to 2020 Financial Year [as of January 29, 2020].

Australia is currently regarded globally as one of the most seriously affected bushfire regions. Over the past 110 years, more than 260 bushfires have resulted in 825 deaths comprising both civilians and firefighters [24,25]. Despite this, while more than 78 % of the fatalities occurred within 30 m of the bushland, 64 % of civilians deaths may be traced to the top 10 worst bushfires in Australia [24]. Historically, Australia's fatality rate is 1 death per 21 homes destroyed by bushfire, while it is 1 death per 13 homes considering 2009 Black Saturday [37]. In contrast, the US's fatality rate is 1 death per 40 homes historically and 1 death per 320 homes considering the 2007 Southern California bushfire [37]. The bushfire fatality rate in Australia is thus higher than in the US. At least 65 bushfire deaths occurred in Australia over the past ten financial years [23]. Notably, over half of the total deaths that occurred during the 2020 financial year, a staggering 54 % comprising of 35 fatalities, exceed the combined total for the previous years (Fig. 1).

Unlike many other at-risk countries, Australia has a regular annual bushfire experience. Despite the lessons learned from previous catastrophic bushfires and preparations for bushfires, bushfire proofing Australia is proving to be challenging to accomplish [38]. While a more significant proportion of bushfire fatalities occurred closer to forests [39], people in general from over 90 % of locations lived within 100 m forests. While many were not directly affected, about 94 % of NSW residents (7.4 million people) were subjected to smoke inhalation of delicate particulate matter [20]. Bushfires accounted for 40 % of deaths arising from natural disaster in Australia since 1900 [7]. The impact of bushfires is enormous and unfortunately, fatality rates are often a result of residents failing to evacuate in a timely manner during catastrophic bushfires [7,9]. Out of 60 % of householders that planned evacuation as their survival strategy on code red (forest fire danger index, FFDI ≥100) during the 2009 Black Saturday bushfire, only 2 % eventually did [7]. Similarly, in a survey conducted by Anton and Lawrence [31] in Western Australia, only 12 out of 300 residents indicated their intention to leave as soon as they knew there was a fire in their area. This implies that many people should include evacuation in their response plans. While it is impossible to address all vulnerabilities stemming from bushfire [40,41], paying adequate attention to minimizing the threats where possible is essential [38].

People live in vulnerable locations for several reasons. First, the number of people living in urban-wildland interfaces, where metropolises meet wildland, increases in response to heightened urban expansion. Second, it is often driven by the high housing costs, like the case of Perth in Western Australia [31]. House prices drive homebuyers and low-income earners into the urban-wildland interface (WUI) [31]. Besides, people tend to live in urban-bush interfaces because of the attractiveness of living in a natural environment [42]. Also, some are motivated by what WUI could offer compared to their usual abodes, such as better lifestyle, more affordable housing, and less congestion [31]. However, the motivating factors driving residents to live on urban fringes can differ from the motivations behind amenity migration to rural areas [31]. For instance, low-income earners or homeowners with lower education levels are likely to disregard risks, which increase their vulnerability to bushfire threats during bushfire mitigation decision-making [43].

### 2.4 Evacuation decision-making

Bushfires can have unimaginable consequences. They result in the loss of human lives, well-being and economic costs [1]. Therefore, decision-making is essential for householders' survival [44]. Decision-making applies to residents in communities where evacuation is either mandatory or voluntary. Despite this, there is a dearth of comprehensive information about residents' intentions to either evacuate or remain within or close to their homes during bushfire seasons [45]. This conflicts with the emerging evidence that some householders are considering staying and defending as an alternative to evacuation [46]. There are significant differences in the behaviour of residents concerning evacuation. For example, in Australia, some householders decided to stay home and actively defended their residences while others who favoured evacuation did not [7,47]. The decision to evacuate or remain and defend is influenced by the number of people available to stay and defend, responsibility for vulnerable household members, level of investment in property and responsibility for livestock and pets [48]. Besides, there are challenges like the complexities of behavioural factors such as property destruction, self-injury or aggressive behaviour influencing householders' actions upon receiving warnings [5]. However, safe evacuation of large groups of householders is more challenging as it is often associated with inadequate warning and evacuations required to take place in a short period. The emphasis is on different cues to act, whether official warnings or environmental ones. Risk attitudes and diverse beliefs about a particular response's efficacy might influence different decisions [49]. In making an informed decision, the risks associated with staying to defend property should be accommodated in community bushfire safety programmes [44]. In addition, consideration should be given to householders' preparedness to leave safely because bushfires have numerous impacts on evacuees. For example, more than one evacuation can occur in a bushfire season, as bushfires can occur during daytime or at night (46). Therefore, this study aims to investigate the factors underlying householders' decisions to remain during bushfire threats.

## 3 Research method

**Fig. 2:**
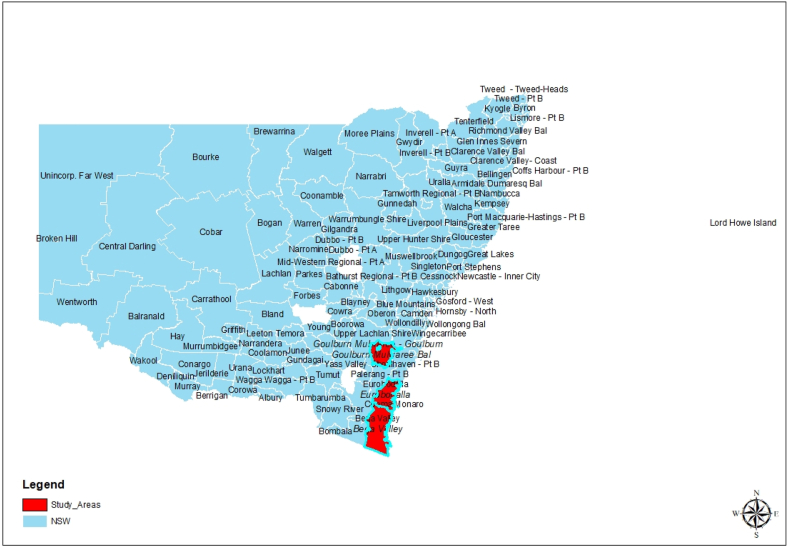
Map of NSW depicting the Study Areas.

Source: Author's Creation (2023)

**Fig. 3:**
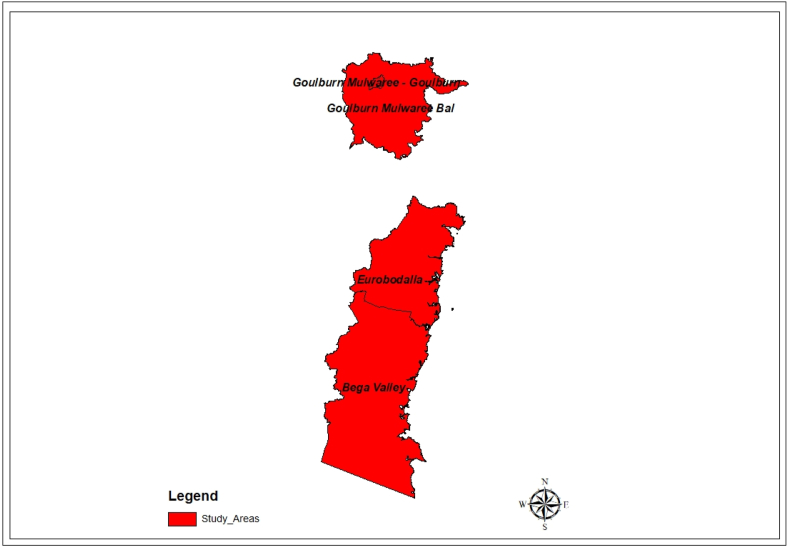
Study Areas (Southeast region of NSW).

Source: Author's Creation (2023)

An inductive research approach was adopted in which the qualitative data collection was carried out using semi-structured interviews with 30 householders. According to Sutton and Austin [50], interviews help researchers to generate information on respondents' views and the meanings behind these views. Moreover, the method was undertaken to clarify, extend, and collect participant feedback. Before conducting the research, ethical approval for the study was given by the human research ethics committee of the University of Newcastle, Australia (Protocol Number H-2021-0284). All participants provided their written informed consent before the interviews. The researchers sent the recruitment flyers to the participants via local councils' newsletters, community Facebook groups, and notice boards. Furthermore, participants from the southeast NSW geographic area were purposively selected (Fig. 2) because the region was the main bushfire-affected area between December 2019 and January 2020 [51]. Householders from three local councils (Fig. 3), Bega Valley Shire, Eurobodalla Shire, and Goulburn Mulwaree Councils, agreed to participate in the study. Interviews were audio recorded and transcribed using Otter.AI software. Participants were referred to using alphanumeric codes rather than their names to ensure confidentiality. So, in this study, the researcher investigated factors underlying householders’ decisions to remain in the Australian bushfire-prone areas from the context of the householders who are survivors of bushfire events. However, the factors identified in the study are not exhaustive or representative of all householders in NSW or Australia, especially when decisions change over time based on new information. The data collection process involved semi-structured interviews conducted both face-to-face and online via the Zoom platform. These interviews took place between April and August 2022, with each session lasting between 40 and 90 min.

Analysis of the transcribed data was guided by the six-phase process for carrying out thematic content analysis of qualitative data [52]. First, the researcher organised and prepared the data to familiarise himself with the data. Second, the data were read carefully to ensure the transcript was understood and a start was made on initial coding. Third, the researcher started searching for themes. Fourth, the researcher reviewed and refined the themes to check if the themes worked at both levels (coded extract, entire data set). Then, the researcher determined how the description and themes would be represented in the results. Finally, the interpretation of the qualitative data was conducted, and the final report produced. Thematic Analysis (TA) is a method for identifying, analysing, and reporting patterns (themes) within data [52]. Further, a foundational approach to analysing raw data was also integrated with a second-stage manual process to conceptualize information into thematic categories [53]. To ensure validity, the interviews were transcribed using Otter.ai software while transcripts were checked manually. According to Sutton and Austin [50] and Owi [54], a researcher's manual checking of transcripts is the first step towards ensuring validity.

Furthermore, constant comparison of developed codes was conducted throughout the analysis to mitigate coding-related errors, thereby enhancing the overall accuracy and consistency of the coding process, as emphasized in previous literature [[55], [56]]. Additionally, inter-rater reliability assessment was undertaken by engaging an independent researcher outside the project team to independently code the de-identified data. This rigorous step aimed to evaluate the consistency and reliability of the coding process. The inter-rater, who is an expert in thematic analysis, meticulously cross-checked the codes for reliability and made necessary adjustments where needed. Moreover, to ensure validation, all transcripts underwent inter-rater reliability measurement, ensuring consistency and reliability across the entirety of the data analysed in the study. This robust methodology, incorporating constant comparison and inter-rater reliability assessment, underscores the study's commitment to rigor and trustworthiness in the thematic analysis process.

The demographic variables such as age, gender, location duration, insurance status, property ownership status, pets' ownership status, and closeness to bushland were collected from householders and subsequently analysed in the study using descriptive statistics. The inclusion of these demographic variables is based on previous research suggesting relationships between these variables and human behaviour in response to bushfires. For instance, Anton and Lawrence [2] reiterated that home ownership could imply owners’ tendency to stay on their properties because of their attachment to them. Another study showed that a significant proportion of bushfire fatalities occurred close to forests because people from over 90 % of locations lived within 100 m of bushland areas [39]. In addition, the decision to evacuate or remain to defend is influenced by the level of responsibility for livestock and pets [48]. So, these previous studies informed the use of demographic variables in this study to understand similarities or otherwise in findings based on these variables [2,39,48]. Furthermore, the interview guide questions were meticulously developed through a collaborative process involving input from the research team and relevant stakeholders, which included a comprehensive review of existing literature. Based on this review, a preliminary set of questions was drafted, focusing on eliciting rich data from participants. The draft underwent a meticulous refinement process involving iterative reviews and pilot testing with six carefully chosen participants. These individuals, though not part of the main study, were selected randomly and shared key demographic similarities. Notably, they reside in regions prone to bushfires and have firsthand experience as survivors of events like the devastating 2019-20 bushfires. This rigorous process resulted in a finalized version of the draft, now accessible in the supplementary materials accompanying the manuscript. Importantly, these participants were not recruited through the same channels as those in the main study. However, they were briefed that they were engaging in pilot testing of the interview protocol, aimed at pinpointing any potential challenges or ambiguities in the data collection process.

This study has several limitations for consideration when interpreting the findings. The small sample size of 30 participants may affect the precision and robustness of the interpretation of the results in the context of diverse demographic factors. It may not represent the views of all those living in bushfire-prone areas. Additionally, the study is limited by a skewed population which was predominantly older (over 55 years) so fewer young people could share their views. While the nine grouped factors provide a comprehensive framework, it is acknowledged that new information may emerge, necessitating adjustments or expansions to enhance the depth of analysis conducted. However, this concern was mitigated by selecting participants with prior bushfires experience despite limited time and resources for data collection influencing the decision to use this sample size. Moreover, it is also essential to note that the small sample size allowed for in-depth exploration of participants' experiences and perspectives. In addition, data saturation was achieved in the study, indicating that sufficient data was collected to capture this specific group's key themes and insights [57]. Notwithstanding the qualitative depth of the study, the small sample size may have restricted our ability to detect subtle variations and perspectives beyond this cohort. Therefore, obtaining more data and conducting further statistical analyses could contribute to a more realistic and nuanced understanding of the implications of these findings about gender dynamics and demographic variations. The geographic areas investigated were those considered bushfire-prone in New South Wales (NSW), Australia. They were the most severely affected by the 2019/2020 bushfires in terms of both the land areas burnt and fatalities. We collected data before the official start of the bushfire fire season (October 1, 2022), to avoid subjecting householders to further trauma.

## 4 Findings and discussion

### 4.1 Demographic information of the interviewees

Table 1 shows the demographic information for the participants. Most were aged 55–74 years, accounting for 80 % (n = 24) of the total population. Further, 17 % (n = 5) were between 35 and 54 years, while the remaining 3 % (n = 1) fell between 18 and 34 years of age. On average, participants were 60 years of age. Most of the participants, accounting for 60 % (n = 18), identified as female, while the remaining 40 % (n = 12) identified as male. Table 1 also reveals the participants’ home and contents insurance status, with 76 % (n = 23) having full insurance coverage. However, 17 % (n = 5) did not have insurance, and 7 % had partial cover. In addition, while 93 % (n = 28) of the participants were homeowners, the remaining 7 % (n = 2) were rentals/leaseholders. While 80 % (n = 24) of the interviewees had pets or animals, 20 % (n = 6) had none. On average, they lived within 59 m of bushland.

**Table 1:** Demographic information of the interviewees.

Classification	Category	Frequency (n)	Percent (%)
**Participants Age**	18–34 years	1	3.33
	35–54 years	5	16.67
	55–74 years	24	80.00
	*Mean Age = 59.88 years*		
	Total	30	100.00
**Gender**	Male	12	40.00
	Female	18	60.00
	Total	30	100.00
**Insurance Status**	Full cover	23	76.67
	Partial cover	2	6.67
	None	5	16.67
	Total	30	100.00
**Property Ownership**	Homeowners	28	93.33
	Renters/Leaseholders	2	6.67
	Total	30	100.00
**Pets Ownership**	Yes	24	80.00
	No	6	20.00
	Total	30	100.00
**Bushland Closeness**	<30 m	9	30.00
	30–50 m	5	16.67
	51–75 m	7	23.33
	76–100 m	3	10.00
	>100 m	6	20.00
	*Mean Closeness = 59.17* *m*		
	Total	30	100.00

### 4.2 Factors influencing decision to remain during bushfires

Table 2 shows 36 factors influencing householders' decisions to remain during bushfires. This study classified the 36 factors into nine distinct groupings, a pivotal measure in organizing our findings cohesively and improving the practical usefulness of our study. Primarily, this organization aspired to elucidate the intricate network of determinants to remain, aligning with a recurring theme in existing literature, where similar variables are frequently grouped to facilitate comprehensive analysis, for example in Adedokun et al. (46). Secondly, these classifications were based on inductive analysis, embracing an inductive approach firmly rooted in the participants' experiences, ensuring the credibility and depth of our categorization process. Thirdly, we conducted rigorous thematic analysis using NVivo 12 Pro, leveraging the software's capabilities to systematically identify patterns and recurring themes in the interview data. This meticulous approach ensured the thoroughness of our categorization. Notably, we attained data saturation after analysing the thirtieth interviewee, marking the point where no new information or themes emerged. The analysis was carried out alongside the interviews being undertaken. This milestone underscores the exhaustive nature of our categorization, reflecting the thoroughness of our analysis and the completeness of our discoveries. The categorization process was transparent and systematic, guided by shared characteristics and thematic resemblances among variables, thus supporting the replicability and validation of our work. Variables were classified based on frequency and prevalence across interviews, allowing us to emphasize consistent themes and significant variations. This categorization process seamlessly complements the overall structure of our research paper, presenting each category with clear and meaningful labels that accurately represent the grouped variables, ultimately contributing to a deeper comprehension of householders' decisions of staying during bushfires. More than previous studies like Whittaker et al. [47] and Strahan et al. [58], the present study presents emerging factors like residents fearing contracting COVID-19, issues surrounding development approval, increased building supplies costs, and clearing post-fire debris.

**Table 2:** Factors influencing householders’ decisions to remain during bushfires.

Child Nodes	References	Parent Nodes	Ref. Total
Protection of properties	13	Protection-Related Factors	32
Protection of animals	8		
Protecting irreplaceable items	4		
Fearing property looters	4		
Protecting forests & wildlife	3		
Self-efficacy to bushfires	8	Attitude-Related Factors	30
Rendering humanitarian services	7		
Attachment to properties	9		
Bushfire threat denial	3		
Unpreparedness towards bushfires	3		
Inaccessible bushfire information	5	Information-Related Factors	28
Inaccurate evacuation information	8		
Untimely bushfire information	6		
Unawareness about bushfire information	3		
Unavailable bushfire warning	6		
Inaccessible support services	6	Operation-Related Factors	22
Denied return access	5		
Service station issues	4		
Lack of inter-agency collaboration	4		
Unavailability of RFS assistance	3		
Road blockade	11	Road-Related Factors	19
Unavailable escape routes	7		
Perceived gridlock	1		
Inadequate evacuation centres	11	Shelter-Related Factors	18
Overcrowded evacuation centres	4		
Lack of places to seek refuge	3		
Lack of insurance	6	Finance-Related Factors	15
Residents' financial difficulty	5		
Loss of tourists' refunds	2		
Difficulty claiming insurance	2		
Development approval issues	7	Rebuilding-Related Factors	13
Increased building supplies cost	5		
Clearing post-fire debris	1		
Elderly health issues	6	Health-Related Factors	10
Fearing contracting covid-19	2		
Trauma in seeking support	2		

#### 4.2.1 Protection-related factors

Table 3 shows the number of codes generated concerning why 57 % (n = 17) of the participants resolved to stay in at-risk bushfire communities, and the number of times referenced. The data show that the participants wanted to protect their valuables, including properties, animals, and irreplaceable items like memories, photos, and artifacts. In addition, three of these participants chose to protect forests and wildlife in their area. Some of the participants said:

*“… at that time, like, we just bought our first house. So, there was no way I was going to just let it burn down while it has the potential of burning down was gonna fight no matter what …”* P1.

*“… I know of a man that stayed. And I think he stayed because he loves his cars. So, he stayed, because obviously he couldn’t drive, I think he’s got three or four, you know, fancy cars. And he couldn’t, which they’re not insured. I don’t think so he couldn’t obviously drive all of them out. So, I think he stayed, he stayed his house burnt down, but he saved the shed with these cars in it …”* P1.

**Table 3:** Protection-related factors.

Child Nodes	Sources	References	Parent Node
Protection of properties	11	13	Protection Related Factors
Protection of animals	7	8	
Protecting irreplaceable items	2	4	
Fearing property looters	2	4	
Protecting forest & wildlife	2	3	
Participants	**17 out of 30**		

However, aside from the impact of bushfires on properties, a few other participants recounted their experience of how looting of properties had prevented them from leaving their properties. One of the participants noted:

*“… my brother decided to stay and defend my property, not only for the fires, but for the looters. Okay. Because and that was what was going around on Facebook. Well, was high, there was a white car with a station wagon going up and down our street, driving very slowly, be careful. So, and that’s what was happening. It was that cars were driving round to look for properties to break into and steal and there was theft. There was theft going on in the middle of all this …”* P17.

Regarding protection-related factors, it became evident that participants' decisions to stay during bushfires were influenced by their concerns about the safety of their properties. This concern was particularly pronounced among 71 % (n = 12) of female participants, highlighting a gender-specific perception of property protection during bushfires. The fact that 71 % (n = 12) of female participants expressed these concerns suggests that there may be gender-based variations in how individuals assess the protection of their properties in such situations. Female participants appeared to attach greater importance to property safety as a factor influencing their decisions to stay. Additionally, the presence of pets played a role in participants’ decisions to stay during bushfires. Most of those who remained on their properties had pets to care for. For instance, 82 % (n = 14) of the seventeen participants that stayed on their properties had pets whilst 18 % (n = 3) did not. This highlights the interconnectedness of protection-related factors, as participants may have stayed not only to safeguard their properties but also to ensure the safety of their pets. Therefore, these participants attributed part of the reasons they stayed to protection-related factors regardless of their ages.

The desire to protect valuables emerged as a compelling factor influencing residents to remain in bushfire-prone communities, even when facing significant risks. According to McLennan et al. [14], those intending to stay and defend were motivated by the desire to protect their property, believed in the success of their efforts, and did not see themselves as risk-takers. In the same vein, the interviews with participants confirmed that some stayed to safeguard their properties, animals, and irreplaceable belongings, aligning with McLennan and Wright [11] and McLennan et al. [14]. These previous studies noted that residents’ desire to protect their homes, assets, and households was a deterrent to evacuating in the presence of bushfires. To prepare their properties for potential fires, these participants engaged in proactive measures such as wetting down buildings, filling downpipes with water, clearing gutters, and reducing fuel loads around their homes [49]. However, it is essential to acknowledge that most buildings are neither designed nor constructed to withstand bushfires, let alone support active defense efforts on days with a catastrophic bushfire danger rating [6]. Furthermore, a significant portion of the buildings belonging to the individuals interviewed were situated along the path of potential bushfires, with 80 % of these structures located within 100 m of bushland, rendering them highly vulnerable to the threat of bushfires.

#### 4.2.2 Attitude-related factors

Forty percent of the total participants exhibited five characteristics grouped into attribute-related factors, as shown in Table 4. The participants were attached to their properties, so they would not leave during bushfires, let alone make somebody else fight for their houses. This attachment could be because some of the participants were in denial of the threats, implying why another set of them were unprepared for the bushfire crisis. While Table 4 shows the number of codes and the number of times referenced, one of the participants’ said:

*“… the other thing is, they are attached to their possessions. You know, they have spent decades sometimes generations, you know, building up a business building up a farm, you know, they feel like it’s part of them that they can’t sacrifice. I’ve never really been like that myself. Of course, now I don’t actually have anything much more like that …”* P24.

**Table 4:** Attitude-related factors.

Child Nodes	Sources	References	Parent Node
Attachment to properties	7	8	Attitude Related Factors
Self-efficacy to bushfires	5	7	
Rendering humanitarian services	4	9	
Bushfire threat denial	2	3	
Unpreparedness towards bushfires	2	3	
Participants	**12 out of 30**		

Other participants said:

*“… and we may not even be able to live back in the areas that we love. Because of that. I think that there’s an old culture of stay and defend, particularly in bush and regional areas …”* P25.

However, while some of the interviewees mentioned they could defend their properties in a bushfire crisis, few others felt they were less susceptible to bushfires in their communities. So, they assigned roles such that they offered help to the highly susceptible members of the community by accommodating them, looking after them in a crisis, and rendering various forms of assistance. For instance, one of the participants said:

*“… That sort of, you know, worry and stress and people coming and people staying and people needing assistance. And we opened our house every time and had people staying here for virtually five weeks. If I had gone and locked up my house, and taken my dogs, and gotten somewhere safe, which I couldn’t do at the time, but if I had, then there would have been another five or so dogs with nowhere to go with their owners. So, in doing what I did, I was able to help more people …”* P19.

The data shows that 40 % (n = 12) of participants exhibited attitude-related factors. Furthermore, age emerged as a potential influencer of attitude-related factors. Older participants displayed attitude-related factors not observed among their younger counterparts. This raises the possibility that age shapes participants' attitudes, reflecting a more ingrained perspective prevalent in bush and regional areas over time. The participants' attributing their decision to stay to the “age-long attitude in bush and regional areas” underscores the complex interplay between cultural norms, generational perspectives, and attitudes towards bushfires. It suggests that attitudes related to bushfire response may be deeply rooted in the cultural and regional contexts in which individuals reside. Furthermore, the presence of pets intersects with these attitude-related factors. Despite being conscious of bushfires, most participants who chose not to evacuate had pets they kept. For instance, about 83 % (n = 10) of the twelve participants had pets, whereas 17 % (n = 2) did not have pets. This suggests that some participants’ attitudes towards pet care may have influenced their decision to stay during bushfires.

The attitude of residents plays a significant role in influencing their decision to remain on their properties, a finding consistent with prior research. For some residents, the longevity factor in their properties was a notable contributor, as they had lived there for extended periods and developed a deep attachment to their homes. This attachment to their properties aligns with the observations of Anton and Lawrence [2], who highlighted that homeowners are more likely to engage in mitigation efforts and stay on their properties due to their emotional connection to them. Furthermore, literature has consistently shown that residents’ affection for community, family, and friends can strongly influence their decision to remain in at-risk areas [59]. Other attitude-related factors that many participants displayed included self-efficacy and a tendency to deny the bushfire threat, contributing to a lack of preparedness. These attitudes are also consistent with findings from previous studies [58,60]. Similarly, according to McLennan et al. [48], the study found that the majority of those who stayed did so out of their commitment to this course of action and prevalence of uncertainty about the threat level in the moments leading up to the fire. In another instance, some residents took on roles such as caregivers, assisting highly vulnerable individuals [59].

#### 4.2.3 Information-related factors

Sixteen out of thirty participants, representing about 53 %, mentioned information-related factors comprising five sub factors as shown in Table 5. The participants stated that information from the rural fire service regarding evacuation was inaccurate. Some residents felt the information was untimely. For instance, some of the participants said:

*“… With the information coming from the New South Wales Government and the Rural Fire Service in Sydney. The Rural Fire Service is controlled by people in offices in Sydney. The information coming from them was outdated. It was incorrect. It was off key. It was wrong in so many ways. And confusing …”* P7.

*“… Many people woke up expecting to get up early and evacuate and the fire had already gone past their house, or their house was already on fire. So that evacuation information was somewhat misleading. But I understand that in catastrophic situation, things can go wrong. But it did allow some people into maybe a false sense of security …”* P20.

**Table 5:** Information-related factors.

Child Nodes	Sources	References	Parent Node
Inaccessible bushfire information	5	5	Information Related Factors
Inaccurate evacuation information	7	8	
Untimely bushfire information	5	6	
Unawareness about bushfire information	3	3	
Unavailable bushfire warning	5	6	
Participants	**16 out of 30**		

In addition, some of the participants noted that bushfire warning messages were not available or that they were unaware of bushfire information. Further, few others wanted to look up information from the council and other emergency agencies’ websites; however, these sources were inaccessible because the fire had destroyed power and internet towers, preventing them from accessing information that could enable them to decide. Other participants said:

*“… And we didn’t really get any warning that it was going to happen. The day before the bushfire hit our house on the bushfire app, it didn’t even say that our house was in the like the Ember zone. So, it wasn’t expected, I suppose …”* P1.

*“… The Internet services when they’re available in advance, are great. After that, you know, electricity went down. Internet went down. SMS went down, mobile services went down. They were erratic, they would come up, they would go down Optus would be on Optus would be off Telstra would be on Telstra would be off …”* P20.

Information-related factors were influenced by gender among the participants, with a notable difference between female and male in terms of their reliance on and perception of information during bushfire crisis. Specifically, 69 % (n = 11) of female participants, who cited ineffective information communication from emergency agencies as a reason for staying during bushfires, suggests that they placed greater importance on receiving accurate and timely information to inform their decisions. In contrast, 31 % (n = 5) of male participants expressed this concern. This underscores the critical role of information in influencing residents’ decisions and actions during disasters. Female participants may have been more reliant on external sources of information, such as emergency agencies, to make informed choices about evacuation and property protection. In contrast, male participants may have relied on other factors or sources of information. Furthermore, the fact that most participants, regardless of gender, had pets, highlights the additional consideration of pet welfare during evacuation decisions.

While information plays a critical role in disaster as it influences residents’ decisions and actions [5], the receipt of information or warnings about bushfire may delay protective action decisions [61]. This delay could stem from the necessity to accomplish a procedure of data exploration and evaluation or as an outcome of contending or conflicting formal or informal data or admonitions, encompassing safe refuge or escape routes [61]. The data analyses show that participants could not access accurate and timely bushfire information resulting in them living under a sense of false security. This corroborates McLennan and Cowlishaw [9], Thompson and Haigh [62], who reiterated that inadequate clear information and untimely messaging influence the residents to remain in at-risk communities. While some residents received incorrect or late bushfire information, a few participants needed help getting bushfire information. These findings align with Blanchi et al. [39] and Whittaker et al. [47], emphasizing that inability to receive an early warning, for example, in cases of rapid onset or communication failure, prevents residents from evacuating safely. The royal commission report also highlighted communication breakdown among residents because bushfires burnt down powerlines and telecommunication towers [63].

#### 4.2.4 Operation-related factors

Twelve out of thirty participants observed that the operation of emergency responders could influence participants to remain, as shown in Table 6, including the number of codes generated and the number of times referenced. Some of the participants noted that the police denied the residents return access to their properties in the previous bushfires. However, these residents believed they could still save their properties from burning down. For example, one of the residents said:

*“… The isle towards town, the road was blocked off and people couldn’t get back to their houses. And there was a couple of houses that could have been saved if people were allowed to come back in and defend them after the main fire went through …”* P5.

**Table 6:** Operation-related factors.

Child Nodes	Sources	References	Parent Node
Inaccessible support services	4	6	Operation Related Factors
Denied return access	5	5	
Service stations issues	3	4	
Lack of inter-agency collaboration	2	4	
Unavailability of RFS assistance	3	3	
Participants	**13 out of 30**		

On the other hand, few others noted that renters stayed on their rental properties because of the non-existence of support services for them if they should lose their rental property to bushfires. One participant said:

*“… I know one circumstance where a man was renting a house, his circumstance could happen to anyone. He was renting a house at a location. Now he was renting it from a friend. And he paid his rent every week. He became homeless because of that bushfire because he lost his rental property. But he couldn’t get any support. Because he didn’t lose his home in the bush fire. He lost his rental property in the bush fire. So, there’s zero support for them. Because the owner of the property couldn’t get any relief or anything. Or he wasn’t he was further down at least on the insurance the house was insured. But because the owner of the house didn’t live in the house, guess what? Any of these absolute weird, crazy situations where renters had to fight to get their bond back because he didn’t leave the house in the state that it was found …”* P2.

Being inadequately resourced to fight fires impacted on the operations of the rural fire service; the participants also observed a lack of inter-agency collaboration among emergency services responders like the NSW rural fire service, fire and rescue, state forests, and marine parks, among others. A participant stated:

*“… in Neira at McDonald’s, or the fire and rescue from Sydney who had tankers down there were at McDonald’s having breakfast because they weren’t allowed to come into the area. So became a bit of a power struggle between the RFS and fire and rescue. If a house is burning, it doesn’t matter whether you’re the RFS or Fire and Rescue said simple. And it’s also about saving lives …”* P2.

One participant's view, regarding the operations of emergency services, is highlighted below:

*“… Like they’re gone like one fellow got the sack here. He was our best. He worked for the State Forest. Because of the disagreements between state forests and marine parks like the marine parks came and said, well, we have done hazard reduction there’s ash going into the water. Now they fined the state forestry $20,000. And this marvellous man got the sack, and they made them sandbags, three kilometres a riverfront, so the ash didn’t go in the water. And it was just, just, it was just ridiculous. And so, we’ve lost that man that knew how to burn it. And the hazard reduction that he was doing was good. It was gentle. And then when the big fire came through the river filled up with ash anyway, like it’s, you know, lack of understanding, I think. Yeah …”* P4.

However, some participants could not leave because their local petrol service station was closed, and when it eventually opened, a power outage affected its operation. One of them said:

*“… down here in Narooma even though we had three petrol stations. It mostly everybody was in four-wheel drives, or diesel cars. And the diesel had been kept for SES and emergency services people. Though the people that were wanting to leave one they couldn’t get out because the highway was cut. But if the highway opened, which it did, they were told they could get over the snowy mountains to Coomer, and then out that way to Canberra, Sydney, and Melbourne. But the thing was there was no service station between Narooma and Coomer. So, they had to have a full tank of diesel before they could leave town. So, they had to wait two days before the service stations were allowed to open …”* P19.

Female participants, constituting about 62 % (n = 8), appeared more prominently influenced by operation-related considerations than their male counterparts, who comprised 38 % (n = 5) of the sample. This gender-based difference could be a variable in understanding residents' responses to operational factors during bushfire situations. Among the operation-related factors discussed, the participants’ desire to return to their properties after the passage of a fire front was a common theme. This suggests that participants, particularly females, may have been more inclined to stay and defend their properties or monitor the situation closely if they believed they would have the opportunity to re-enter their communities after the immediate danger had passed. Additionally, the presence of pets among a substantial portion of the participants (77 %, n = 10) highlights the importance of considering the welfare and safety of animals in evacuation plans and operations. Participants who owned pets may have been more hesitant to evacuate if they perceived challenges or lack of provisions for their animals in emergency response operations.

The operation-related factors examined in this study pertain to the activities of emergency responders and authorities. The finding that some participants believed the police would deny them access to their properties is consistent with the observations of Leonard and Bowditch (64), who noted that a significant number of houses destroyed in bushfires initially survived the passage of the fire front but later succumbed to flames ignited by flying embers a few hours later. This suggests that householders could have saved their properties had they been allowed to return and extinguish spot fires. Furthermore, the lack of assistance from the Rural Fire Service (RFS) in supporting communities aligns with findings from previous studies [20,46]. The Emergency Leaders for Climate Action [64] report highlights the under-resourcing of emergency services, land management agencies, and fire services due to the federal government's underestimation of the increasing threat of climate change. Service stations also hindered residents' ability to evacuate, either due to the need for additional fuel to accommodate the increased demand or because they were unable to dispense fuel due to a loss of electricity needed to power the pumps.

#### 4.2.5 Road-related factors

Eleven out of thirty participants, representing 37 %, narrated three factors grouped into road-related factors in Table 7 including the number of codes generated and the number of times the factors were referenced. First, most of the participants noted road blockades could prevent residents from leaving, therefore, they stayed. For instance, one of the participants said:

*“… The highway, the Princes Highway was closed so all traffic had been diverted past our home. And it was like bumper to bumper, no traffic was moving. It was just like a car park. Sydney traffic miles just said. And my son rang me to say why aren’t you leaving? And I said, because there’s no point in leaving because we couldn’t go anywhere. We can’t even get out of our driveway. I said we will be leaving tomorrow morning. But we’re not going with this crowd. Because we felt the crowd was as big an issue as …”* P16.

**Table 7:** Road-related factors.

Child Nodes	Sources	References	Parent Node
Possible road blockade	8	11	Road Related Factors
Unavailable escape routes	7	7	
Perceived gridlock	1	1	
Participants	**11 out of 30**		

Second, a few participants mentioned the lack of escape routes if the Princess Highway had been closed. The participants could not leave their communities during the crisis except to stay and defend them. Some of them said:

*“… Well, we couldn’t leave the town. Yeah, we couldn’t leave here because there were fires all around us. So, you couldn’t leave where we were …”* P15.

Another participant said:

*“… Because for quite a long part of that, we couldn’t get out anyway. Okay, that we only had we could only stay in defence, we couldn’t go anywhere …”* P19.

In the third instance, a few others mentioned that road gridlock could make them stay rather than leave and get stuck on the road, thereby exposing them to more dangers than if they were at home. One of the participants’ views is highlighted below:

*“… if I knew that every road leading out of the bay was going to be gridlocked, and I was going to be stuck for five hours in my car, um, then I, you know, that was the other thing …”* P17.

The study's findings reveal how participants perceived and responded to road-related factors as constraints to evacuating from bushfire-prone areas. Female participants, constituting a significant majority at 82 % (n = 9) of the group, reported being concerned by road-related factors than males amounting to 18 % (n = 2). Interestingly, these participants who were constrained by road-related factors may have been particularly affected by road-related issues when deciding whether to evacuate, possibly due to experience, mobility, or familiarity with their communities.

Road-related factors significantly contributed to residents’ decision not to evacuate from at-risk areas. Notably, concerns about potential road blockades and the perceived likelihood of traffic gridlock played a role in dissuading householders from evacuating. This finding aligns with existing literature, underscoring the impact of road closures and inaccessible access routes in impeding swift egress during bushfires [20,39]. Many participants described their locations as having only one road leading in and out of their communities. In such scenarios, safe evacuation becomes nearly impossible when bushfires compromise the sole road servicing the community. Furthermore, only one access route poses challenges for emergency responders in assisting at-risk communities and efficiently supplying relief materials to residents [47].

#### 4.2.6 Shelter-related factors

Eight of thirty participants, representing 27 % (n = 8), mentioned three factors that were grouped into shelter-related factors in Table 8 including the number of codes generated and the number of times the factors were referenced. Further, most of these participants noted that evacuation centres where residents could seek refuge during bushfires were inadequate for users. For instance, one of the participants who would have evacuated said:

*“… in that situation, there wasn’t enough room at the evacuation centre and I feel that maybe like the school hall or you know another, there could have been another centre that could have accommodated some people but there wasn’t any there like would have been hard to do because yeah, like there wouldn’t have been any bedding or things like that so yeah, so they had the evacuation centre they had all the hotels in the area full because it was already full anyway from all the tourists that come down and then but then maybe they needed like an another place for people to go as well …”* P15.

**Table 8:** Shelter-related factors.

Child Nodes	Sources	References	Parent Node
Inadequate evacuation centres	6	11	Shelter Related Factors
Overcrowded evacuation centres	3	4	
Lack of places to go	3	3	
Participants	**8 out of 30**		

However, few other participants noted that the available evacuation centres were overcrowded and without adequate facilities. The participants could not leave their communities during the crisis because of overcrowding. Some of them said:

*“… and nearest evacuation centre there’s too many people for the one evacuation centre. It’s, it’s like a central evacuation centre that services many, many people so and then, because where we live, it’s a holiday area. So, in January, you can have up to I don’t know 100,200 times more people than you would normally have. And that’s what happened in the black summer fires. So, you have all the people who are down here for holidays, as well as those who are living here. So, it’s like our areas like ours that have high visitation during the high-risk summer periods. Almost need to bushfire plans, one for managing during the January high season period, and one for one for other times …”* P15.

Aside from residents that went to the evacuation centres despite their inadequacy, some went to friends and family's houses. However, few participants did not have places they could go to during the crisis; therefore, they stayed on their properties. The participant said:

*“… And she kept saying to him, stop buying so much stuff. We’ve got nowhere to go and nowhere to put it. I don’t know what we’re going to do. So, stop buying so much stuff …”* P19.

Another participant said:

*“… We have the resources to think, oh, well, we’ll just go and book into a motel where it’s safe for a while, you know, we can do that. But in the high season, everything’s booked out, so you have nowhere to go. And you can’t stay in a tent because the smokes too thick …”* P26.

All 8 participants reporting shelter related factors were female. Shelter-related concerns were raised by participants across various age groups, with a notable exception of one participant aged between 18 and 34 years. Interestingly, in this age group, shelter-related factors were not explicitly mentioned. It raises the question of whether individuals in this demographic had alternative reasons for staying during a disaster or if shelter concerns were not their primary factor. Regarding insurance coverage, participants revealed they had either full or partial home and contents insurance for their properties. This finding suggests that a certain level of insurance coverage was typical among participants, potentially influencing their decision to stay during a disaster. Furthermore, it is noteworthy that most participants (75 %, n = 6) who mentioned shelter-related concerns also had pets that they kept on their properties, thus implying that pet ownership could substantially influence their decision to stay.

The role of shelter during a disaster like a bushfire is significant. Therefore, an alternative source of shelter or a place to seek refuge influences residents' decisions and actions. Almost half of the participants did not evacuate because of inadequate evacuation centres or a lack of places to seek refuge from disaster. This finding is in tandem with the Owens and O'Kane [20] and Commonwealth of Australia [63] that reported inadequacy and not-fit-for-purpose concerning evacuation centres. Most evacuation centres were overcrowded and lacked adequate facilities like toilets, water, bedding, and high efficiency particulate air (HEPA) filters. Further, the centres and facilities did not meet the needs of the disabled and older people. Similarly, an earlier study also emphasized the state of evacuation centres and their facilities influencing residents' to remain on their properties during bushfires [46].

#### 4.2.7 Finance-related factors

Eleven out of thirty participants, representing 37 %, noted that finance-related factors influenced participants to remain. Table 9 shows the number of codes generated for the factors and the number of times referenced. Besides, lack of insurance, residents' financial difficulty, difficulty in claiming insurance, and loss of tourists’ refunds were four factors grouped into finance-related factors. First, some participants lacked home and contents insurance because they could not afford it. For instance, one of the participants who would have evacuated said:

*“… I stayed. I was on my own just with my dog. We had too much valuable stuff that wasn’t insured because I don’t have a lot of money, so we had to stay and fight …”* P4.

**Table 9:** Finance-related factors.

Child Nodes	Sources	References	Parent Node
Lack of insurance	6	6	Finance-Related Factors
Residents' financial difficulty	4	5	
Loss of tourists' refund	1	2	
Difficulty in claiming insurance	1	2	
Participants	**11 out of 30**		

Further, another set of participants were not financially capable (to transport their belongings, buy petrol to fuel cars, purchase food items and other essentials) of undertaking evacuation because they never expected to have to do this. For example, one of the participants said:

*“… And that was another thing when all the power went down on New Year’s Eve. On that very first morning. None of the ATMs worked. So, nobody could get cash. And you go to the shop, but none of the ATMs worked. So, you couldn’t pay for food because you didn’t have any cash. And it was just an ongoing thing, the same as the petrol stations. Because the power was out, the whole system fell over. And with the amount of people reliant on cards now and not carrying cash in cash, what happens in that instance?”* P19.

However, a few participants (8 %) mentioned their inability to finance the rebuild of their properties because of difficulties associated with insurance claims. They noted that many still lived in a caravan almost three years after the 2019/2020 bushfires. One participant said:

*“… when people who have insurance, make a claim. The insurance company often puts a lot of obstacles in the way of paying the claim, you know, or they’ll say, you’re underinsured. We give you this much, or you made such and such a modification without our approval, blah, blah, blah, blah, blah. I have just heard people talking about problems they’ve had. The reason why they’re still living in a caravan is because they can’t get the money from the insurance to rebuild …”* P24.

Besides, the tourists felt tourism management would not refund their money, so they resolved to stay in at-risk communities rather than leave when emergency responders sent evacuation messages. One such participant said:

*“… Well, you know, the problem with the tourists was they paid they were scared they weren’t going to get their money back and they weren’t going to get their money back. So, they put their money before their families …”* P23.

More female participants (73 %, n = 8) responded to finance-related factors as a constraint to evacuation than males, who accounted for 27 % (n = 3). Moreover, finance-related factors were mentioned across all age groups of participants, indicating that financial difficulties were not specific to a particular age range but affected individuals of varying ages. Most participants who mentioned finance-related factors (82 %, n = 9) had full or partial insurance coverage for their properties, which may have provided some financial security during the disaster. However, it is worth noting that 18 % (n = 2) of the participants did not have home and contents insurance, particularly leaseholders. This lack of insurance could have contributed to their financial difficulties during the evacuation. Interestingly, a significant portion of the participants (73 %, n = 8) who cited finance-related factors as a constraint also had pets they kept on their properties. This suggests that the financial burden of evacuating with pets may have been an additional challenge for these individuals, potentially influencing their decision to stay. The data strongly indicates that financial difficulties posed a significant barrier to evacuation for many householders. This implies that financial constraints were widespread issues faced by the participants, such as the inability to refuel vehicles and pay for hotel accommodations. The unprecedented nature of the 2019/2020 bushfires likely exacerbated these challenges.

This focus on residents' financial constraints is noteworthy, as previous studies, such as Adedokun and Egbelakin [46], did not report such a factor. It is important to note that the earlier study was based on a literature review. Furthermore, some participants faced challenges affording insurance for their properties due to financial difficulties. Even for those with insurance, the inability to secure the necessary funds for reconstruction work discouraged them from evacuating during subsequent bushfires. These findings are consistent with the observations of Tibbits and Whittaker [65], who emphasized that a lack of, or inadequate, insurance played a significant role in householders’ decisions not to evacuate from bushfires. Few tourists considered in the study chose not to evacuate when advised to do so because they were aware that they would likely be unable to recover the money they had already paid for tourism activities. This implies that tourists risk exposure to disasters and potentially endanger the lives of emergency responders, such as firefighters, who might be called upon to assist these tourists in hazardous conditions.

#### 4.2.8 Rebuilding-related factors

Table 10 shows that 23 % (n = 7) of participants stated that rebuilding-related factors influenced their decisions to stay. The rebuilding-related factors include development approval issues, increased building supplies cost, and clearing post-fire debris. Table 10 also shows the number of codes generated for rebuilding-related factors and the number of times they were referenced. Generally, five out of these seven participants noted that residents had problems sorting out council development approval (DA) and construction certificates. They mentioned several untended DAs in the council, which could be one of the reasons why some residents sold their land and moved on or waited to fight bushfires to save their properties. The untended DAs could lead to a rebuilding time lag. For instance, one of the participants said:

*“… There were still DAs in front of Council of people who are trying to rebuild. They’ve noticed some people sold their land and moved to Sydney moved up to the north coast. I know one couple they lost everything Yes, they end up buying a new home and selling their land because the DA procedure was just so frustrating and depressing and emotional …”* P2.

**Table 10:** Rebuilding-related factors.

Child Nodes	Sources	References	Parent Node
Development approval issues	5	7	Rebuilding Related Factors
Increased building supplies cost	5	5	
Clearing post-fire debris	1	1	
Participants	**7 out of 30**		

Further, participants stated that residents wanted to rebuild their lost properties in the aftermath of bushfires; however, the cost of building supplies had increased significantly from when they built their buildings. The significant increase in building supplies discouraged several residents from undertaking evacuation so they could defend their properties and avert rebuilding works. A typical example is when one of the participants said:

*“… I’ll tell you what happened in a lot of instances because we’re builders, we have worked in fire zones before. What happens is when a fire goes through and decimates an area if that area hadn’t already been given a BAL rating, it is afterwards. And those houses that are destroyed might have been insured for, let’s pull a number out of the hat 500,000. But because of the new BAL rating,*^1^*it’s going to take another 100 to 200,000 to rebuild that house. So, your house is gonna take 700,000 to rebuild. So, they don’t have enough money to build their house that they had before. So, then they have to cut back and cut back and reorganize, to work out what they’re gonna do or organize a loan. And you know, what the banks are like at the moment …”* P19.

Besides, the owners bear the costs associated with removing the property remains, except in few instances where there are interventions from politicians and the state government. Therefore, to avoid incurring the cost of removal of building remains in the event of bushfires, the householder stayed on their properties in defence. One participant said:

*“… the State Member for further down south said, guess what everyone, I have negotiated with New South Wales government, whether you were insured or not, you were going to remove all the remains of your home at no charge. This is to help you. Yep, that’s really nice …”* P2.

More female participants (57 %, n = 4) presented rebuilding-related factors than males (43 %, n = 3). Moreover, rebuilding-related factors were reported by all participants except for the age group aged 35–54 who did not mention it. It raises the question of why this particular age group exhibited a different response, warranting further investigation. Regardless of gender or age, most participants had full or partial insurance coverage for their home and contents, potentially providing financial support for rebuilding. However, it is noteworthy that 29 % of the participants did not subscribe to home and contents insurance coverage, especially among the leaseholders. This lack of insurance coverage could have significantly contributed to the concerns related to rebuilding, particularly for those without insurance. Interestingly, 57 % of the participants who presented rebuilding-related factors also had pets they kept on their properties. This suggests that the presence of pets added complexity to their rebuilding considerations and potentially influenced their decision to stay.

Participants in this study preferred staying to defend their properties rather than allowing them to be destroyed by bushfires. This inclination is rooted in the perceived difficulty and protracted process of securing development approval from local councils for rebuilding efforts. This finding aligns with Prelgauskas [66], who observed that the post-fire reassessment of hazard-level criteria could introduce a new set of development requirements no longer compatible with previous standards, resulting in a complex and time-consuming approval process. Furthermore, the increased cost of building supplies, often exceeding the average financial capability of householders, added to the challenges of reconstruction. Additionally, damaged properties required demolition and removal, incurring additional costs before reconstruction could commence. These findings echo the observations made by Prelgauskas [66]. As a result, the likelihood of delays in commencing reconstruction work is high. Unresolved issues related to rebuilding factors could deter householders from being willing to self-evacuate during future bushfire events if these challenges are not addressed.

#### 4.2.9 Health-related factors

Table 11 shows that older people's health issues, fear of contracting COVID-19, and trauma in seeking support were three factors grouped into health-related factors based on eight of thirty participants, representing 27 %. In addition, most of the participants discussed mobility issues, people who had dementia among older people, and people in slings requiring assistance to get out of their beds. For instance, one of the participants said:

*“… And the elderly couple had mobility issues that they really couldn’t go to an evacuation centre. So, I helped them with private accommodation in Bermagui, so that they could go somewhere that would be safe. That they could stay in comfort and have their own bathroom and things like that. So, they didn’t have to be in a community of many people …”* P16.

**Table 11:** Health-related factors.

Child Nodes	Sources	References	Parent Node
Elderly health issues	5	6	Health Related Factors
Fearing contracting covid-19	2	2	
Trauma in seeking support	2	2	
Participants	**8 out of 30**		

Further, a few others feared contracting COVID-19 from the crowd in the evacuation centre and other airborne diseases. A typical example is when one of the participants said:

*“… since March 2020, my concern has been okay couple of things. Since the bushfires, many people in our area have developed respiratory problems, so from breathing bushfire smoke for so long, and my husband one of them. So is at high risk if he gets COVID. So, with a pandemic, and you’ve got like this traditional evacuation centre that has like in it hundred thousand of people all together, and there’s like COVID risk. Yeah, that’s not a good situation to me, and you’re like, take your life out of one place and put it in another. So yeah, I guess moving forward, having COVID safe evacuation is important …”* P26.

Moreover, another set of participants felt constrained to request help or assistance from charity organizations or government support services. So, they remained on their properties to defend it because they could not go through the trauma of seeking support if bushfires impacted them. One of the participants from a charity organization said:

*“… I heard that a lot from people that they found it traumatizing to walk in and ask for help. Because they’ve never had to ask for help before in their life. And I know a couple of people said it took them four or five times to actually get enough gumption or nerve to actually walk in the door and start the process …”* P24.

Most female participants (87 %, n = 7) presented factors influencing their decisions to remain, whereas males accounted for only 13 % (n = 1). Furthermore, health-related factors were mentioned by participants aged 55–74 years. This suggests that individuals in this specific age group were more likely to mention health-related concerns as factors influencing their decision to stay during the disaster. In addition, most of the participants (87 %, n = 7) who presented factors influencing their decisions to remain on their properties had entire home and contents insurance coverage for their properties. However, it is essential to note that 13 % (n = 1) of participants did not subscribe to insurance for their properties. This lack of insurance coverage may have added to the complexity of their decision-making, especially in the context of health-related concerns. Interestingly, 63 % of the participants who presented health-related factors also had pets they kept on their properties. This suggests that the presence of pets contributed to their decision to stay due to health-related factors. It is evident from the findings that among the householders in communities at-risk of bushfires are older people, whose health issues constrained them to remain in place. This finding is consistent with previous studies indicating that older people, people with disabilities, or vulnerable people are not able to evacuate from bushfire-prone areas [9,47,65]. The transcripts show that some older people in the community had dementia while others were in slings and required assistance. In addition, some householders never thought of evacuating to evacuation centres primarily because of their fear of contracting COVID-19. The trauma of seeking support from not-for-profit organizations or evacuation centres discouraged several householders from evacuating. These two factors are emerging and not found in previous studies before the 2019/2020 bushfires.

## 5 Conclusions and implications

### 5.1 Conclusions

The study contributes new insights by examining diverse factors influencing householders' decision-making during bushfires, including protection-related aspects, such as safeguarding valuables and concerns about looting, alongside gender-specific perceptions and considerations for pets. These contribute to the complexity of decision-making and enrich existing literature on evacuation behaviours during crises. The findings provide a holistic understanding crucial for developing targeted strategies and advancing knowledge in disaster management and community resilience. Moreover, attachment to properties, cultural influences, community responsibilities, and pet care further influence attitudes, highlighting the intricate web of considerations residents navigate. In addition, the study also reveals that information-related challenges, operational factors, road-related issues, shelter concerns, and financial constraints are integral components of residents' choices during bushfires. Participants’ experiences with police denial, limited support for renters, and operational hindrances like local petrol station closures add complexity to evacuation decisions. Road-related challenges, including blockades, congestion, and fear of gridlock, underscore practical barriers to leaving.

Furthermore, inadequacies in evacuation centres, overcrowding, and limited shelter alternatives present logistical challenges, further influencing residents' choices. Finance-related factors, such as insurance coverage, unexpected burdens, and difficulties in claims, underscore the financial considerations shaping evacuation decisions. Additionally, rebuilding-related issues, including bureaucratic hurdles and increased costs, contribute to the overall complexity of decision-making. Health-related concerns, particularly for older people, fear of COVID-19, trauma associated with seeking support, and the intersection with pet care, reveal additional dimensions influencing residents’ decisions. Considering these multifaceted factors, the study underscores the need for tailored strategies for timely evacuation. Moreover, considering these could lead to early self-evacuation among householders in future bushfires, ultimately improving the safety and well-being of residents in bushfire-prone areas, including overall community resilience.

### 5.2 Implications for research

In the context of future research on early self-evacuation from bushfires, it is imperative to include an examination of financial factors. These financial factors entail a comprehensive exploration of the pivotal role of financial considerations, encompassing aspects such as insurance affordability, the expenses associated with evacuation, and the financial constraints residents' encounter. Moreover, researchers should investigate the diverse demographics within at-risk communities. This scrutiny should extend to demographic variations in evacuation decisions, considering factors such as gender, age, and the presence of pets. Understanding these demographic nuances is crucial for tailoring effective evacuation strategies and crafting targeted messaging. Furthermore, an in-depth exploration of the challenges associated with post-fire rebuilding is warranted. This inquiry should analyse the protracted development approval process and the financial burdens accompanying reconstruction efforts. By shedding light on these challenges, research can illuminate their impact on residents' evacuation choices. Additionally, it is essential to delve into the behaviour of tourists during bushfire events. Tourists represent a distinct demographic whose decisions not to evacuate can have significant consequences for emergency responders. Research in this realm should delve into the motivations and barriers that shape tourists’ choices in evacuation scenarios, contributing valuable insights for emergency management and preparedness.

### 5.3 Implications for practice

These findings underscore the need for emergency management agencies to consider several key strategies. First, providing financial assistance to residents for evacuation-related expenses could serve as a potent incentive for early self-evacuation. Transparency in financial support mechanisms motivates residents to leave high-risk areas. Second, ensuring the accessibility of up-to-date and accurate information, particularly regarding evacuation routes and shelter options, is essential in promoting timely evacuations. Third, addressing the needs of pet owners by prioritizing the availability of pet-friendly evacuation facilities could encourage a broader segment of the population to evacuate. Fourth, streamlining the post-fire rebuilding process, reducing delays in development approval, and exploring options for affordable building supplies is imperative to alleviate residents' concerns and encourage evacuation without fear of reconstruction challenges. Fifth, community education programs should emphasize the importance of early self-evacuation and address residents’ specific concerns. Tailored messaging strategies should be employed to reach different demographic groups within the community effectively. Lastly, raising awareness among tourists about the risks of remaining in disaster-prone areas and the importance of heeding evacuation advisories could reduce resource strain and minimize risks during emergencies.

## Data availability statement

Data will be made available on request.

## CRediT authorship contribution statement

**Olufisayo Adedokun:** Writing – review & editing, Writing – original draft, Software, Resources, Methodology, Investigation, Formal analysis, Conceptualization. **Temitope Egbelakin:** Writing – review & editing, Supervision, Software, Resources, Methodology, Investigation, Formal analysis, Conceptualization. **Willy Sher:** Writing – review & editing, Supervision, Software, Resources, Methodology, Investigation, Formal analysis, Conceptualization. **Thayaparan Gajendran:** Writing – review & editing, Supervision, Software, Resources, Methodology, Investigation, Formal analysis, Conceptualization.

## Declaration of competing interest

The authors declare that they have no known competing financial interests or personal relationships that could have appeared to influence the work reported in this paper.

## Footnotes

1 *Bushfire Attack Level (BAL) is a means of measuring the severity of a building's potential exposure to ember attack, radiant heat, and direct flame contact, expressed in kilowatts/m2*.
