# Supplementary material for: Investigating factors underlying why householders remain in at-risk areas during bushfire disaster in Australia
Source: Heliyon. 2024 Apr 16;10(8):e29727. doi: 10.1016/j.heliyon.2024.e29727 (PMC11053178; doi:10.1016/j.heliyon.2024.e29727)
Supplement: Multimedia component 1 [file mmc1.docx]

**Research Working Title: Incentives for Encouraging Early Self-Evacuation During Bushfire Crisis**

**Semi-Structured Interview guide**

**Interview Protocol**

Introduce self

Introduce research & explain purpose

Provide ethics statement

Confidentiality

Obtain consent

If consent is obtained, proceed with the interview

| **Demographic Background of the Interviewees** | | |
| --- | --- | --- |
| **Date:** | **Time:** | **Place:** |
|  | | |
| Age  18 – 34  35 – 54  55 – 74  over 75 | How long have you been living in your current place?  < 5 years  5 to 10 years  11 to 15 years  16 – 20 years  > 20 years | What is your home & contents insurance status?  Full cover  Partial cover  None |
| Do you own or rent your property?  Homeowners  Renters/leaseholders | Do you have pets or animals?  Yes  No | What gender do you identify with?  Male  Female  Others specify |
| What is your property type?  Residential block  Small acreage/hobby farm/lifestyle block  Working farm  Large acreage/lifestyle | How close is your home to bushland?  < 30 metres  30 ≤ × ≤ 50 metres  51 ≤ × ≤ 75 metres  76 ≤ × ≤ 100 metres  > 100 metres | Would you be available for follow-up interview questions?  Yes  No |

**Bushfire Scenario**

Now imagine that during the fire season, you and all those who normally reside with you are at home. It has been declared a day of “ Catastrophic Fire Danger”, and there is a Total Fire Ban for your region of the State. At about 3 pm you become aware of advice to evacuate (on the radio, or a web site, or by email, or text, or telephone) that there is a large bushfire burning out of control and it will probably hit your location in 1 -2 hours. You look outside and see a large plume of smoke being blown toward your property.

1. **Following the bushfire scenario, kindly answer the below questions by ticking the appropriate box where 5 = very high; 4 = high; 3 = average; 2 = low and 1 = very low**

| **S/N** | **Questions** | **5** | **4** | **3** | **2** | **1** |
| --- | --- | --- | --- | --- | --- | --- |
| 1 | What is the likelihood of bushfire occurring in your community? |  |  |  |  |  |
| 2 | What do you think the impacts of a large out of control bushfire could be in your community? |  |  |  |  |  |
| 3 | What would be your level of concern for a large out of control bushfires in your area? |  |  |  |  |  |
| 4 | How vulnerable are you or your property/residence to the threats of a large out of control bushfires? |  |  |  |  |  |
|  |  |  |  |  |  |  |

1. **Kindly tick the appropriate box if you are aware or unaware of the support services you could get should you need to evacuate for bushfires .**

| **S/N** | **Support Services** | **Aware** | **Unaware** |
| --- | --- | --- | --- |
| 1 | Immediate financial assistance |  |  |
| 2 | Emergency accommodation |  |  |
| 3 | Emergency catering |  |  |
| 4 | Personal support |  |  |
| 5 | Material aid |  |  |
| 6 | Disaster relief grant |  |  |
| 7 | Multi-faith chaplaincy |  |  |
| 8 | Accessible routes |  |  |
| 9 | Others specify... |  |  |
| 10 |  |  |  |
| 11 |  |  |  |
| 12 |  |  |  |

**Ice-breaking questions**

1. Tell me, how did you and your family come to live in this area?
2. Since you have lived here, can you share your experience of bushfires?
3. What have you learnt about bushfire seasons in this area?

| **S/N** | **Research Objectives** | **Interview Questions** |
| --- | --- | --- |
|  |  |  |
| 1a | **To examine how residents perceive bushfire threats** | Tell me why you think bushfires are likely or not likely to happen in your community |
|  |  | Why do you think the impacts of bushfires are likely to be very high in your community? |
|  |  | Why are you concerned or not so concerned about the threats from these bushfires? |
|  |  | Tell me why you think you and your property/ residence might be vulnerable to the threat of a large out of control bushfires? |
|  |  | **END OF THE SCENARIO** |
|  |  | **GENERAL EVACUATION QUESTIONS** |
| 1b | **To examine how residents respond to bushfire threats** | **- Past:** Tell me what you did in the last bushfire crisis? E.g., stay or leave early  Why did you stay or leave early?  **-Future:** If there were to be another large out of control bushfires, tell me what you would do the same or differently and why? |
|  |  | Tell me about your ability to leave early when advised to do so.  Why do you think you could or could not do this? |
|  |  | How important do you think early self-evacuation is to prevent people dying in bushfires? |
|  |  | How important do you think early self-evacuation is to prevent mental health impacts? |
|  |  | As a resident, under what conditions would you self-evacuate early? |
|  |  | How would you react to a compulsory evacuation order? |
| 2 | **To identify factors influencing residents’ decisions to engage in at-risk behaviour** | **Information factors** |
|  |  | Thinking about the bushfires you have experienced, please tell me:  What information/messages from **RFS** did you get? |
|  |  | What do you feel is helpful /unhelpful with the current bushfire risk information? |
|  |  | How has the information/messages received from RFS influenced your evacuation decisions? |
|  |  | Is the current approach working? If not, why is this so? |
|  |  | How do you think bushfire risk information can be improved? |
|  |  | Who can help improve it? |
|  |  | **Operational Factors** |
|  |  | Tell me about other institutional support or lack of support (LEMC, Red Cross, Blaze-Aid, Anglicare, Salvation Army) during bushfire crisis.  How has the institutional support you received influenced your evacuation decisions? |
|  |  | Could you describe where your closest emergency evacuation area is? |
|  |  | Have you heard of a Neighbourhood Safer Place, and do you know what it means? |
|  |  | Do you find the term Neighbourhood Safer Space confusing? If yes, how? |
|  |  | What concerns do you have around evacuating? |
|  |  | How much will these concerns influence your future evacuation decisions? |
|  |  | Tell me about your awareness of the local conditions and locations.  How has the awareness or lack of awareness of local conditions and locations influenced your evacuation decisions? |
|  |  | **Social factors** |
|  |  | When would you consider it is too late to evacuate safely?  How would it influence your evacuation decisions? |
|  |  | Please tell me about the influence of community on your evacuation decisions. |
|  |  | How successful do you think you’d be in defending your house?  How does this influence your evacuation decisions? |
|  |  | Tell me how safe you think you and your family are in your house when bushfire threatens.  How will this influence your evacuation decisions? |
|  |  | **Protection factors** |
|  |  | Tell me about things that will make you not to evacuate |
|  |  | How will they influence your evacuation decisions? |
|  |  | Tell me about how your home and its contents influenced your evacuation decisions. |
|  |  | Tell me about your concern for your pets/animals.  How does this influence your evacuation decisions?  Tell me more about the available support services for people evacuating with animals  How does this influence your evacuation decisions? |
|  |  | What influence does insurance of home and contents have on your evacuation decisions? |
|  |  | **Operational factors** |
|  |  | Are there any time you wanted to evacuate but you didn’t due to possible road closures?  Please tell me how possible road closures could influence your evacuation decisions? |
|  |  | Have you thought about the facilities that you would need to evacuate to? What do you think you would need? Do you think these would be available? |
|  |  | Sometimes support centres are in different states. If you had to cross a state border, would this influence your evacuation decisions? |
|  |  | Before you can access support services (accommodation, catering services, material aid), you will be required to give personal information to authorities. If different services asked you for the same information, how would you feel? |
|  |  |  |
|  |  | How does the current ‘Prepare, Act, Survive’ influence your evacuation decisions? |
|  |  |  |
| 3 | **To investigate incentives that could encourage residents to self-evacuate** | Thinking about your awareness of support services, tell me about the influence of these services on your evacuation decisions? |
|  |  | What would encourage you to self-evacuate early?  Why are they important to you? |
|  |  | What can the government and private sector do to improve your decisions about evacuating early? |
|  |  | Finally, is there anything else you would add? |
